# Diffusion of DNA on Atomically Flat 2D Material Surfaces

**DOI:** 10.1021/acsnano.4c16277

**Published:** 2025-06-05

**Authors:** Dong Hoon Shin, Sung Hyun Kim, Kush Coshic, Kenji Watanabe, Takashi Taniguchi, Gerard J. Verbiest, Sabina Caneva, Aleksei Aksimentiev, Peter G. Steeneken, Chirlmin Joo

**Affiliations:** † Kavli Institute of Nanoscience Delft, Lorentzweg 1, 2628 CJ Delft, The Netherlands; ‡ Department of Precision and Microsystems Engineering, 2860 Delft University of Technology, Mekelweg 2, 2628 CD Delft, The Netherlands; § Department of Electronics and Information Engineering, Korea University, Sejong 30019, Republic of Korea; ∥ Department of Bionanoscience, Delft University of Technology, 2629 HZ Delft, The Netherlands; ⊥ New and Renewable Energy Research Center, 26717 Ewha Womans University, Seoul 03760, Republic of Korea; # Center for Biophysics and Quantitative Biology, 14589 University of Illinois at Urbana−Champaign, Urbana, Illinois 61801, United States; ¶ Advanced Materials Laboratory, 52747 National Institute for Materials Science, Tsukuba 305-0044, Japan; ∇ Department of Physics and Beckman Institute for Advanced Science and Technology, University of Illinois at Urbana−Champaign, Urbana, Illinois 61801, United States; ○ Department of Physics, Ewha Womans University, Seoul 03760, Republic of Korea

**Keywords:** van der Waals materials, hexagonal boron nitride, DNA, surface diffusion, nanofluidics

## Abstract

Accurate localization and delivery of biomolecules are pivotal for building tools to understand biology. The interactions of biomolecules with atomically flat 2D surfaces offer a means to realize both the localization and delivery, yet experimental utilization of such interactions has remained elusive. By combining single-molecule detection methods with computational approaches, we comprehensively characterize the interactions of individual DNA molecules with hexagonal boron nitride (hBN) surfaces. Our experiments directly show that, upon binding to a hBN surface, a DNA molecule retains its ability to diffuse along the surface. Further, we show that the magnitude and direction of such diffusion can be controlled by the DNA length, the surface topography, and atomic defects. We observe that the diffusion speed of the biomolecules is significantly lower than indicated by molecular dynamic simulations. Through computational analysis, we present the model based on temporary trapping by atomic defects that accounts for those observations. By fabricating a narrow hBN ribbon structure, we achieve pseudo-1D confinement, demonstrating its potential for nanofluidic guiding of biomolecules.

## Graphical abstract

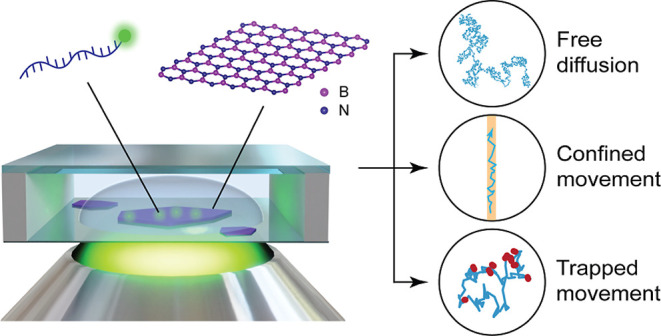

1

The localization of biomolecules such as DNA and proteins is an indispensable endeavor, bearing immense significance in the comprehensive study of biological entities and their interactions.^1–6^ Achieving such localization without the need for tethering or constraining these molecules would not only facilitate the accurate detection and identification of single biomolecules but also empower the direct monitoring and controlling of biochemical reactions and the detailed characterization of their properties.^7–11^ A promising avenue involves characterizing the properties of biomolecules as they traverse a precisely engineered two-dimensional (2D) surface. This surface serves as a nanoscale 2D biocharacterization processing line, enabling the isolation, sorting, and concentration of individual target entities. This approach has the potential to achieve ultimate selectivity and sensitivity.^12–15^

In a recent development toward this aim, simulation studies proposed utilizing the mobility of biomolecules on flat 2D van der Waals material (vdWM) surfaces as a platform for single-molecule detection and manipulation.^16–28^ Several implementations have been proposed, including guiding DNA toward a nanopore by means of a graphene step edge^27^ and linearizing individual DNA and protein molecules by employing heterostructures of graphene/hexagonal boron nitride (hBN)/graphene.^19,20,24^ The vdWMs exhibit a remarkable range of opto-electro-mechanical properties,^29–32^ while also being biocompatible and robust in physiological environments.^25,33–36^ Importantly, vdWMs can be seamlessly integrated into a single device and the large and atomically flat 2D surfaces of vdWMs provide ample space for the study of molecular dynamics and reaction processes, propelling the exploration of vdWMs as a new single-molecule platform in the fields of bioscience and biotechnology.^37–41^

Despite these promising prospects, to date, only few experimental studies have addressed the binding and diffusion of biomolecules on vdWMs at single-molecule resolution.^42^ This pursuit has been hindered by the stringent requirements of the surface, which must possess three key properties: (i) optimal binding energy allowing mobility of biomolecules while ensuring prolonged localization, (ii) chemical stability and high cleanliness in aqueous environments, and (iii) no attributes such as fluorescence quenching or autofluorescence that could compromise optical measurement techniques including single-molecule fluorescence.

We identify hBN as a promising contender as it possesses outstanding chemical inertness and thermal stability^43–46^ whereas its wide energy bandgap of ∼6 eV precludes fluorescence quenching and autofluorescence.^47–50^ Consequently, fluorophore-labeled molecules on a hBN surface could be detected even at the single-molecule level, which was previously unattainable with graphene due to significant quenching effects.^51–53^ In addition, hBN is particularly well suited for studying the kinetics of DNA because the interatomic spacing of B and N atoms in the hexagonal 2D lattice closely matches, within 1.6%, the C–C bond length of the hexagonal rings in nucleobases of DNA.^43,54,55^ Thus, it is expected that the hydrophobic interaction due to these well-matched atomic distances leads to adsorption of DNA molecules. Moreover, it was predicted that graphene and hBN surfaces allow unimpeded lateral motion of molecules, enabling these to easily slide across the surface given the equivalent surface lattice sites.^56^ This characteristic contrasts with more conventional surfaces, such as silicon nitride or silicon dioxide, which are known for their atomic roughness that significantly hinder lateral diffusion.^57^ Despite these advantages, the detection of biomolecules and characterization of their dynamics on hBN surfaces have been limited, primarily due to technical challenges in imaging and a lack of complete understanding of the interactions between these surfaces and biomolecules.

Here, we present a comprehensive study of the binding and diffusion of DNA on hBN surfaces, using a combination of experimental and computational techniques. We find that single-stranded DNA (ssDNA) molecules localize on the pristine, untreated hBN surface, resulting in their confinement within the 2D plane. The ssDNA molecules engage in diffusion on the surface of hBN, permitting us to explore the intricacies of their motion through a single-molecule tracking approach. Importantly, we reveal the role of several defect types on biomolecule motion and demonstrate that step edges and domain boundaries of hBN spatially constrain the motion of the molecules. By engineering a narrow hBN ribbon structure, we further increase the confinement effect and clearly observe the motion of ssDNA molecules along the narrow channel, showcasing the potential application of hBN in nanofluidics and single-molecule transport.

## 2 Results and Discussion

### 2.1 DNA Adsorption on hBN Surfaces

To investigate the interaction between DNA molecules and the hBN surface at single-molecule resolution, we prepared 35 nt-long ssDNA molecules labeled with a Cy3 fluorophore. The hBN surface was prepared on a borosilicate coverslip via the mechanical exfoliation technique (Methods). Since the flakes on the coverslip were freshly cleaved, the topmost hBN surface was nearly contaminant-free. Figure 1a illustrates the wide-field epifluorescence single-molecule microscope used to capture fluorescence images of a hBN flake immersed in a buffer solution carrying the ssDNA molecules.

**Figure 1:**
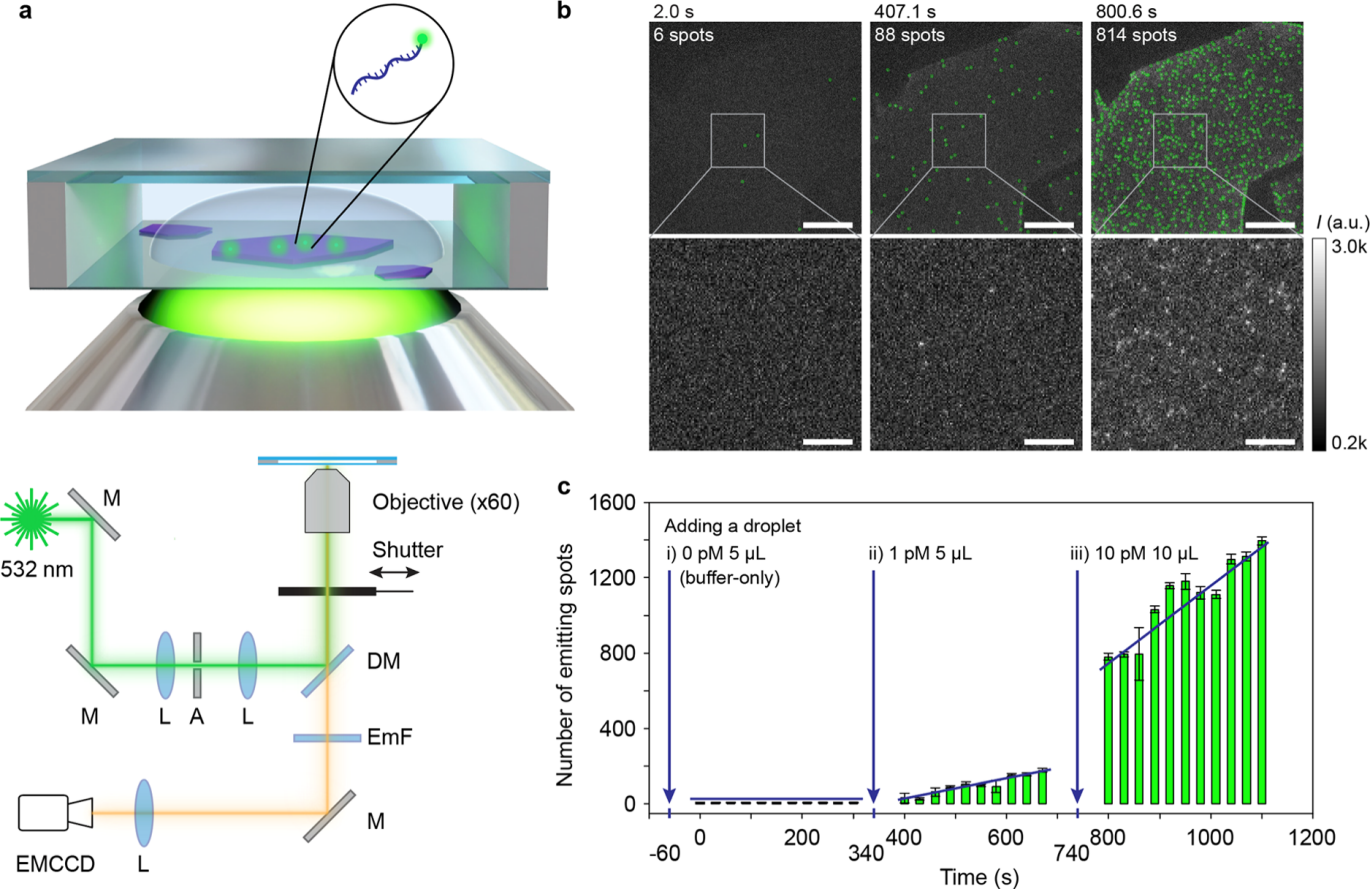
Single-molecule observation of ssDNA on the hBN surface. (a) A schematic representation of the sample chamber and measurement setup. Fluorescently labeled ssDNA molecules (green) adsorbed onto the surface of an hBN flake (purple) are imaged using a wide-field epifluorescence microscopy setup. M, mirror; L, lens; A, aperture; DM, dichroic mirror; EmF, emission filter. (b) Fluorescence microscopy images of Cy3-labeled ssDNA molecules adsorbed on the hBN surface after injection of 0, 1, and 10 pM DNA concentrations (from left to right) at indicated times. ssDNA molecules in the top panels are marked by a green circle. The bottom panels show the magnified view of the areas within the white boxes in the top panel. The scale bars represent 20 μm (top) and 5 μm (bottom), respectively. (c) Temporal evolution of fluorescence emission spots counted on the flake area shown in the top panels of Figure 1b. A 5 μL of droplet containing no DNA, 5 μL of 1 pM DNA and 10 μL of 10 pM DNA were added at −60 s, 340 and 740 s, respectively (blue arrows). The time scale corresponds to that shown above panels in 1b.

We first measured the background signal of the pristine hBN surface by applying a 5 μL droplet of blank buffer solution. We observed ∼1 emitting spot per 1000 μm^2^ on the hBN surface (Figure 1b,c). When we added a 5 μL droplet containing 1 pM ssDNA, we observed that the number of fluorescence spots increased at a constant rate. A higher rate was observed when an additional droplet of 10 pM ssDNA was added, providing evidence that the fluorescence spots correspond to ssDNA molecules.

### 2.2 2D Diffusion of DNA

During the single-molecule fluorescence measurements, it became apparent that the adhered ssDNA molecules moved over the surface, exhibiting substantial surface mobility (Movies S1, S2, S3 and S4), as predicted by previous molecular dynamics (MD) simulations.^20,22–24,27,28^ The motion of the adsorbed ssDNA was notably faster when we used a shorter 7 nt (nucleotide)-long ssDNA instead of the long 35 nt ssDNA (Movies S1 and S3). For a quantitative analysis, we extracted trajectories of individual ssDNA molecules by using a single-molecule tracking algorithm (Figure 2a and Methods). Figure 2b shows the trajectories recorded over 300 s, and Figure 2c shows all the trajectories located within the square area. Note that the surface density of ssDNA was kept below 0.2 μm^–2^, ensuring spatial separation sufficient to prevent interactions and thus minimizing any potential crowding or clustering effects. The 2D diffusion motion of ssDNA molecules on the hBN surface implies the surface binding energy of ssDNA is high enough to localize the molecule near the surface for a prolonged time. Additionally, this suggests a uniform binding energy per atom, allowing the molecules to diffuse along the surface without any energy penalty. Because hBN generally remains hydrophobic and exhibits minimal net surface charge in aqueous conditions,^58,59^ electrostatic interactions with negatively charged biomolecules like DNA are expected to be weak. Consequently, we anticipate only a minor influence of charge effects on adsorption and diffusion behavior of DNA molecules in our experiments.

**Figure 2:**
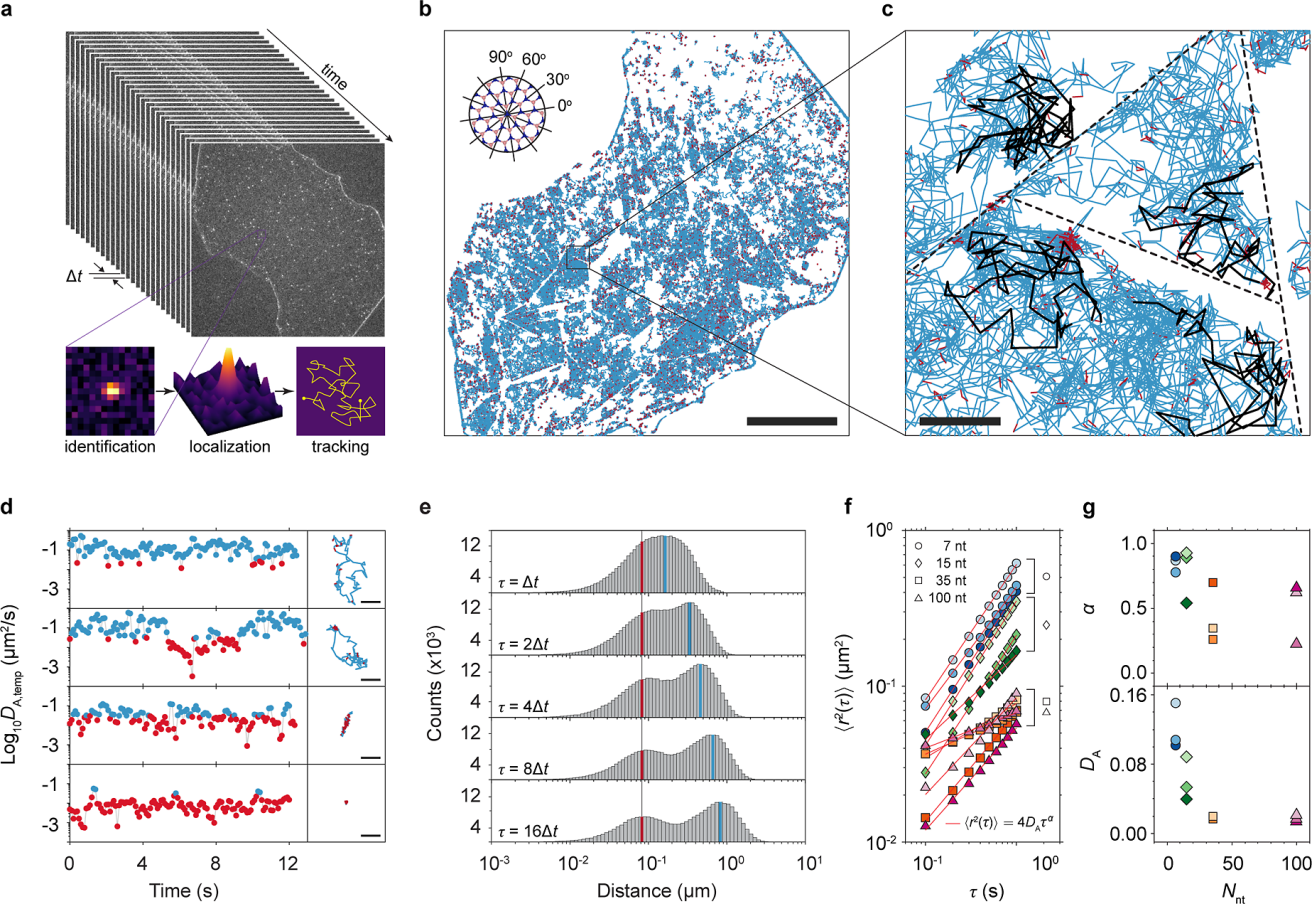
2D diffusion of ssDNA on hBN surfaces. (a) Schematic illustration of the single-molecule tracking analysis methodology. Identification of fluorescent spots in the fluorescence microscopy images were performed using differences of Gaussian (DoG) algorithm combined with a quadratic fitting scheme with subpixel resolution. The localized spot positions were then tracked across consecutive frames to establish molecule trajectories. (b) Trajectories of individual ssDNA molecules (7 nt) recorded over 400 s. Blue and red colors indicate mobile and stationary phases determined by temporal apparent diffusion coefficient (*D*_A,temp_) values (see Figure 2d), respectively (see Methods for details). The lines in the top left corner denote the angles of the hBN lattice determined by the flake edges. The scale bar is 20 μm. (c) A zoom-in image from a square area in (b). The black trajectories are representative trajectories showing the confined movement in domain boundaries defined by step edges (potential location indicated by dashed lines). The scale bar is 1 μm. (d) Time traces of the apparent diffusion coefficient (*D*_A_) of four representative molecules (left panels) with their 2D trajectories (right panels), with blue and red indicating the mobile and stationary states, respectively. The scale bars in the right panels are 1 μm. (e) Jump distance distributions at various lag times τ *= n*Δ*t*, with *n* = 1, 2, 4, 8, 16 and the frame time Δ*t* = 0.1 s. The distributions show two distinct components, mobile (blue) and stationary (red). (f) Mean squared displacement ⟨*r* ^2^(τ)⟩ for ssDNA molecules of different lengths plotted as a function of lag time τ. Data measured from different hBN surfaces are marked with different colors. (g) The apparent diffusion coefficient *D*_A_ and the diffusion exponent α in ⟨*r* ^2^(τ)⟩ = 4*D*_A_τ^α^ are determined through linear fitting of the data in (f), as depicted by the red lines. The fill colors differentiate the data sets, which correspond to those in (f).

The trajectory maps revealed that there were boundaries on the hBN surface that ssDNA molecules were unable to traverse. Most boundaries were straight lines that run parallel to the lattice angles derived from the edge of the hBN flake (Figure 2b inset and Figure S1). This confinement effect aligns well with recent MD simulations on ssDNA movement on graphene.^27^ These simulations showed that the rate at which ssDNA moves across an atomic step is much lower than the rate at which it moves over a flat 2D plane since ssDNA molecules encounter resistance when they attempt to traverse the atomic step. This resistance exists not only at the up-steps but also at the down-steps, confining ssDNA effectively within the boundaries of a flat terrace on the hBN crystal.

Closer analysis of single-molecule trajectories reveals that the diffusive motion of ssDNA molecules is occasionally arrested or slowed down over varying periods of time (red traces in Figure 2b). These stoppages and slow movements were identified based on whether the apparent temporal diffusion coefficient (*D*_A,temp_) fell below a certain threshold value of 0.031 μm^2^/s. These stoppage events occur not only at step edges but also in the middle of a domain. This is more clearly illustrated in Figure 2d,e, where *D*_A,temp_ and jump distances were derived from the trajectory data. Figure 2d presents *D*_A,temp_ plotted over time as well as the corresponding trajectories, with blue and red colors indicating values that are above and below the threshold, respectively. The graphs from top to bottom display four molecules as representative examples, each demonstrating different types of motion: fast continuous diffusion, fast diffusion followed by stoppage, linear diffusion along a straight line and long-term anchoring at a single position. In Figure 2e, the jump distances, the distance that a ssDNA molecule travels during a certain lag time τ, calculated for different values of τ *= n*Δ*t*, with *n* = 1, 2, 4, 8, 16 and Δ*t* = 0.1 s are shown. The histograms show two peaks, suggesting that the motion can be categorized into two states, slow (red line) and fast (blue line) diffusion. As the slow peak does not change its position with increasing lag times, we attribute it as a stationary state of molecules. We note that the jump distances measured from the stationary phase are ∼0.1 μm (red lines in Figure 2e), which is below the optical diffraction limit. Thus, the jump distances from the stationary phase probably merely reflect the uncertainty in the position determination from the single-molecule images and do not necessarily correspond to motion of the molecule. Given our typical photon counts (40–45 counts per 100 ms exposure) and background (∼20 counts), we determined that the associated Cramér–Rao lower bound is 0.096 μm,^60^ indicating that 0.1 μm closely matches the practical resolution limit under our imaging conditions (Methods).

Further experiments using ssDNA strands of different lengths, specifically 7, 15, 35, and 100 nt, show a decrease in both the apparent diffusion coefficient *D*_A_ and the diffusion exponent α values as the length of the DNA strand increases. Three data sets, all of which were measured from different hBN surfaces and are plotted separately in Figure 2g, were collected per length of ssDNA. *D*_A_ and α were determined by fitting the average mean squared displacement (MSD) with the function ⟨*r* ^2^(τ)⟩ = 4*D*_A_τ^α^, where ⟨*r* ^2^(τ)⟩ and τ denote ensemble average of the displacement and the lag time, respectively (Figure 2f).^61^ Longer ssDNA molecules exhibit lower mobility *D*_A_ and more pronounced subdiffusive behavior, i.e. α < 1, indicating anomalous diffusion. Interestingly, *D*_A_ has a clear trend with length with no significant differences between the data sets, while α has a wide margin of error across the different data sets (Figure 2g). This indicates that DNA mobility is primarily affected by its length, while subdiffusivity is influenced not only by the DNA length but also by the local character of the hBN surface, which is sample dependent, e.g. due to variations in the configuration of step edges and the domains they define on the hBN surface.

### 2.3 Computational Study of DNA Diffusion over hBN Surfaces

To elucidate the diffusion mechanism of DNA on the hBN surface, we performed MD simulations using the same parameters used in the experiments, i.e. the same buffer, salts, and the length of the ssDNA. As depicted in Figure 3, three types of hBN surfaces were simulated: a perfect planar surface (Figure 3a), a surface with a step edge (Figure 3b), and a surface containing 0.1% of B and N vacancies (Figure 3c).

**Figure 3:**
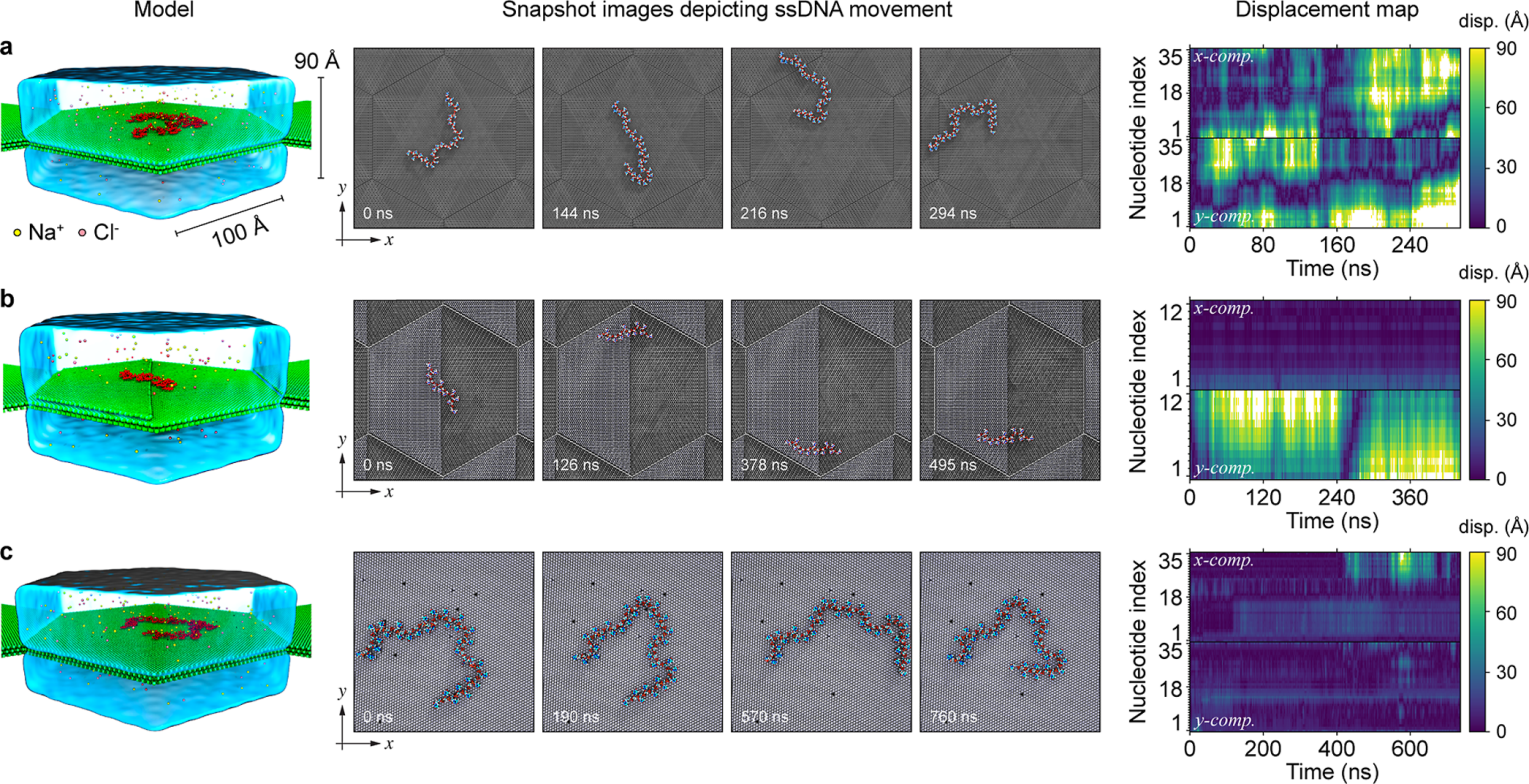
MD simulation of ssDNA diffusion on hBN surfaces. Three distinct types of hBN surfaces were investigated: a perfectly flat surface (a), a surface with a single step edge (b), and a surface with atomic defects (c). The displacement maps on the last column show the displacement in *x* and *y* of each nucleotide as a function of time.

On the perfect planar hBN surface, the ssDNA molecule slid freely as visualized in the snapshot images in Figure 3a and Movie S5. The ssDNA molecules with lengths of 7, 15, 35, and 64 nt exhibited normal 2D diffusion with diffusion coefficients of 749, 228, 111, and 65 μm^2^/s, respectively, as presented in Figure S2. Notably, these values were significantly higher than the experimental values in Figure 2g by a factor of 10^3^–10^4^. The simulation results suggested that the diffusion coefficients are largely independent of the nucleotide type, as supported by the comparison of multiple sequences of 25 nt ssDNA on a perfectly flat hBN surface (Figure S3). The simulations repeated using five different DNA sequences showed qualitatively similar behavior. The MD simulation in Figures 3b and S4 and Movie S6 also provides evidence supporting the possibility of linear movement of a ssDNA molecule along a step edge, similarly to the observation in the third row of Figure 2d. The displacement maps clearly illustrate the differences in DNA dynamics between a perfect planar surface and a surface with a step edge. On the perfect planar surface, high displacements occurred almost continuously in both the *x* and *y* directions. In contrast, on the surface with a step edge, displacements were not only reduced but also predominantly occurred along the *y*-axis, parallel to the step edge.

On the hBN surface containing 0.1% of atomic defects, the motions of ssDNA molecule experienced transient arrests, as illustrated in Figures 3c and S5 as well as Movies S7 and S8. When the ssDNA molecule was bound to multiple atomic defects, it exhibited limited mobility within the group of defects. However, it was also observed that ssDNA was not permanently bound to the defects, and some segments of the molecule could escape from the defects, as depicted in the displacement map.

Our MD simulation revealed how ssDNA molecules interacted with the hBN surface at the nanosecond time scale, yet the large differences in the diffusion coefficients between the experiments and simulations remained to be explained. The MD simulations were limited to the (sub)­microsecond range, whereas the experimental time scale was several hundreds of seconds. To bridge this time scale gap, we investigated the defect-controlled motion observed in our MD simulations using the Monte Carlo (MC) simulation method. We assumed that a freely diffusing molecule could be temporarily trapped on the surface when it encountered a defect site. As illustrated in Figure 4a, the 2D diffusion was modeled with the following conditions: the DNA molecules diffuse on a finite square surface with area *L*_d_^2^, with a free-diffusion coefficient (*D*_0_) obtained from MD simulations of a perfectly flat hBN surface (Methods).

**Figure 4:**
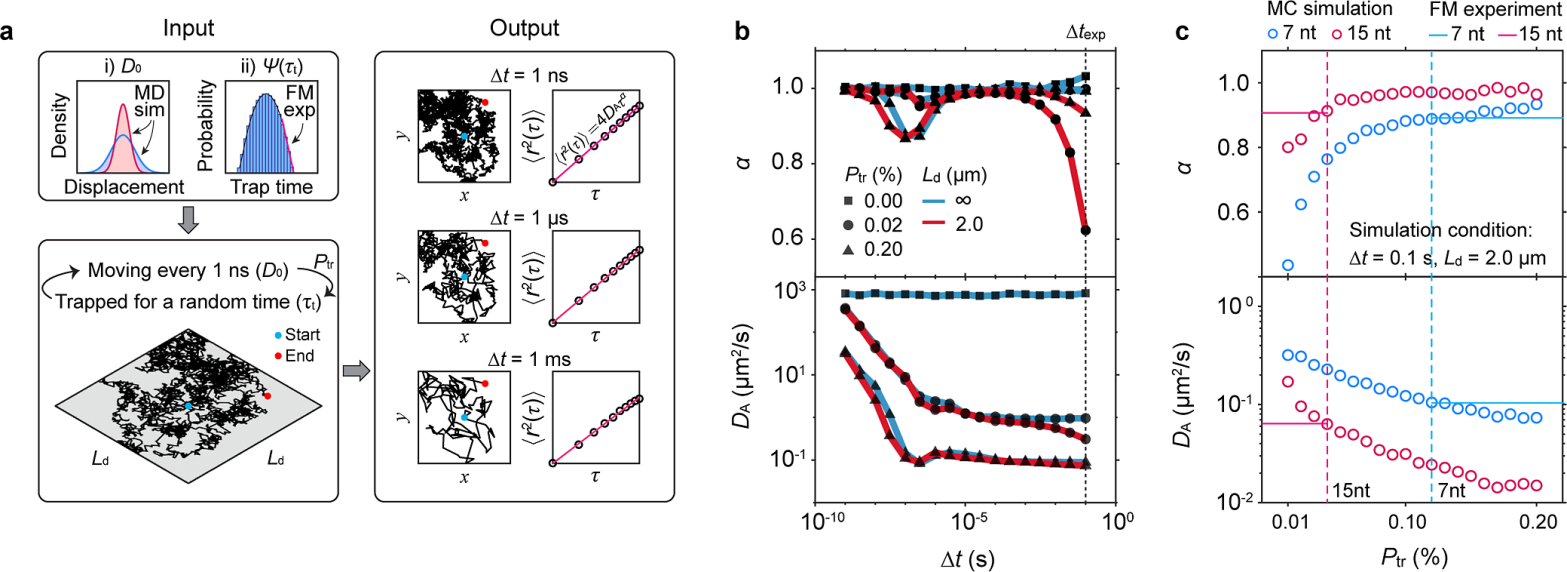
Monte Carlo (MC) simulations of ssDNA diffusion on hBN surfaces. (a) Conceptual outline of the MC simulation model, which employs particles that diffuse, with diffusion coefficient *D*_0_ determined from MD, in simulation steps of 1 ns with probabilities *P*_tr_ per step of encountering trapping sites. Trapped particles remain stationary for a random trap time τ_t_, before resuming movement. This behavior is simulated using the probability distribution function ψ­(τ_t_), derived from single-molecule fluorescence microscopy data (Figure S6). If a particle crosses the domain boundary *L*_d_^2^ based on its calculated displacement, its location is adjusted to be mirrored off the boundary, ensuring it remains within the domain boundary. By fitting the resulting MSDs, the effect of the domain size *L*_d_ and the trapping probability *P*_tr_ on the apparent diffusion coefficient *D*_A_ and the diffusion exponent α are determined. (b) Simulated variations in *D*_A_ and α for various *P*_tr_ and *L*_d_ are plotted as functions of frame time Δ*t* when *D*_0_ = 749 μm^2^/s. The dashed line indicates Δ*t* used in our fluorescence microscopy measurements. (c) Influence of *P*_tr_ on *D*_A_ and α at set conditions of Δ*t* = 0.1 s and *L*_d_ = 2.0 μm, with blue and red lines representing experimental data for 7 nt and 15 nt ssDNA molecules. The corresponding *P*_tr_ values are 0.12% and 0.04% for 7 nt and 15 nt ssDNA molecules, respectively.

The apparent diffusion coefficient *D*_A_ remained constant regardless of Δ*t* when *P*_tr_ = 0, which is the ideal case for normal diffusion with no trapping. In contrast, when *P*_tr_ was greater than zero, *D*_A_ decreased as τ increased. For instance, at *P*_tr_ values between 0.02% and 0.2%, *D*_A_ drastically reduced from 749 μm^2^/s at Δ*t* = 1 ns and then saturated to less than 1 μm^2^/s at Δ*t* = 0.1 s, as depicted in Figure 4b. This implies that transient trapping at atomic defect sites could be responsible for the discrepancy between the MD simulation and experimental results. The diffusion exponent (α) showed two regimes of subdiffusivity, 1 ns < Δ*t* < 10 μs and Δ*t* > 0.1 ms (Figure 4b). While the first regime originates from the trapping rate since it is comparable to Δ*t/P*_tr_, the second regime is attributed to the confinement effect from the step edges at domain boundaries.

We find that for 7 nt and 15 nt ssDNAs, the *P*_tr_ values that match the experiment results are 0.12% and 0.04%, respectively (blue and red lines in Figure 4c). The *P*_tr_ can also be estimated based on the speed of ssDNA diffusion and the density of defects on the hBN surface so that the reliability of the MC simulation results can be verified. For instance, with *P*_tr_ = 0.12%, the 7 nt ssDNA diffusion lifetime is calculated to be τ_d_ = 1 ns/0.0012 = 0.83 μs. Using this lifetime and the MD diffusion coefficient we can estimate the typical diffusion length *l*_d_ of the molecules with the 2D random walk equation $l_{d}=\sqrt{4D\tau_{d}}$ . If this length is limited by trapping by defects, then it should be an estimate for the defect density. In the case of 7 nt ssDNA, we obtained the typical diffusion length *l*_d_ = 50 nm with *D*_0_ = 749 μm^2^/s. For 15 nt ssDNA, we obtained τ_d_ = 2.5 μs and *l*_d_ = 47.7 nm using *D*_0_ = 228 μm^2^/s. The diffusion lengths of the two ssDNA with different lengths were similar to each other, suggesting that it is limited by surface defects. We can estimate the defect density to be approximately (1 cm/50 nm)^2^ cm^–2^ = 4 × 10^10^ cm^–2^, which is consistent with expected values for high-pressure grown hBN flakes.^62–64^

We conclude that the diffusion of ssDNA on the hBN surface has two phases. On a defect-free, flat hBN surface, ssDNA moves with a high diffusion coefficient of e.g. 749 μm^2^/s for 7 nt-long DNA. On a surface with atomic defects, the ssDNA can be intermittently trapped at defect locations. The trapping probability is estimated from MC simulations, from which we estimate a trap density of 4 × 10^10^ cm^–2^ with an average trap time τ_*t*,avg_ = τ_0_ exp­(−μ) = ∼335 μs. The existence of these trapping sites can thus account for the experimentally observed apparent diffusion rates *D*_A_. Notably, a recent study demonstrated DNA immobilization on graphene surfaces synthesized via chemical vapor deposition (CVD).^65^ While the previous study utilized graphene rather than hBN, the observed immobilization was attributed to surface defects. Given that CVD-grown 2D materials generally exhibit higher defect densities than exfoliated flakes synthesized under high-temperature and high-pressure conditions, this observation supports our findings, underscoring the significant role of defect density in DNA mobility on 2D surfaces.

### 2.4 Confinement of ssDNA Molecules in a hBN Ribbon

In Figure 2b, we discussed the confinement effects induced by step edges by limiting the motion of the ssDNA molecules within a single hBN terrace. This characteristic offers the opportunity to develop highly localized channels that can guide DNA in a pseudo-one-dimensional manner. Notably, vdWMs like hBN possess an inherent property of cleaving along the crystal orientation, facilitating the preparation of clean ribbon-shaped crystalline surfaces. To demonstrate the feasibility of guiding molecules, we show in Figure 5 and Movies S9 the motion of a 16 nt ssDNA on a narrow, elongated hBN ribbon extending from a larger region of the crystal. Figure 5a represents a superposition of 3500 images recorded during 350 s, where the letters c-f indicate the motion of a ssDNA molecule, shown in more detail in Figure 5c–f. As seen in Figure 5b, while the apparent diffusion coefficient (*D*_A_ = 0.039 μm^2^/s) value is comparable to that on a large 2D surface, we observe pronounced subdiffusivity (α = 0.64). Figure 5c–f highlight various characteristic ssDNA movements on the hBN ribbon, demonstrating ssDNA entering through an inlet (Figure 5c), moving linearly along the channel (Figure 5d), and navigating through *y*-junction branches (Figure 5e,f).

**Figure 5:**
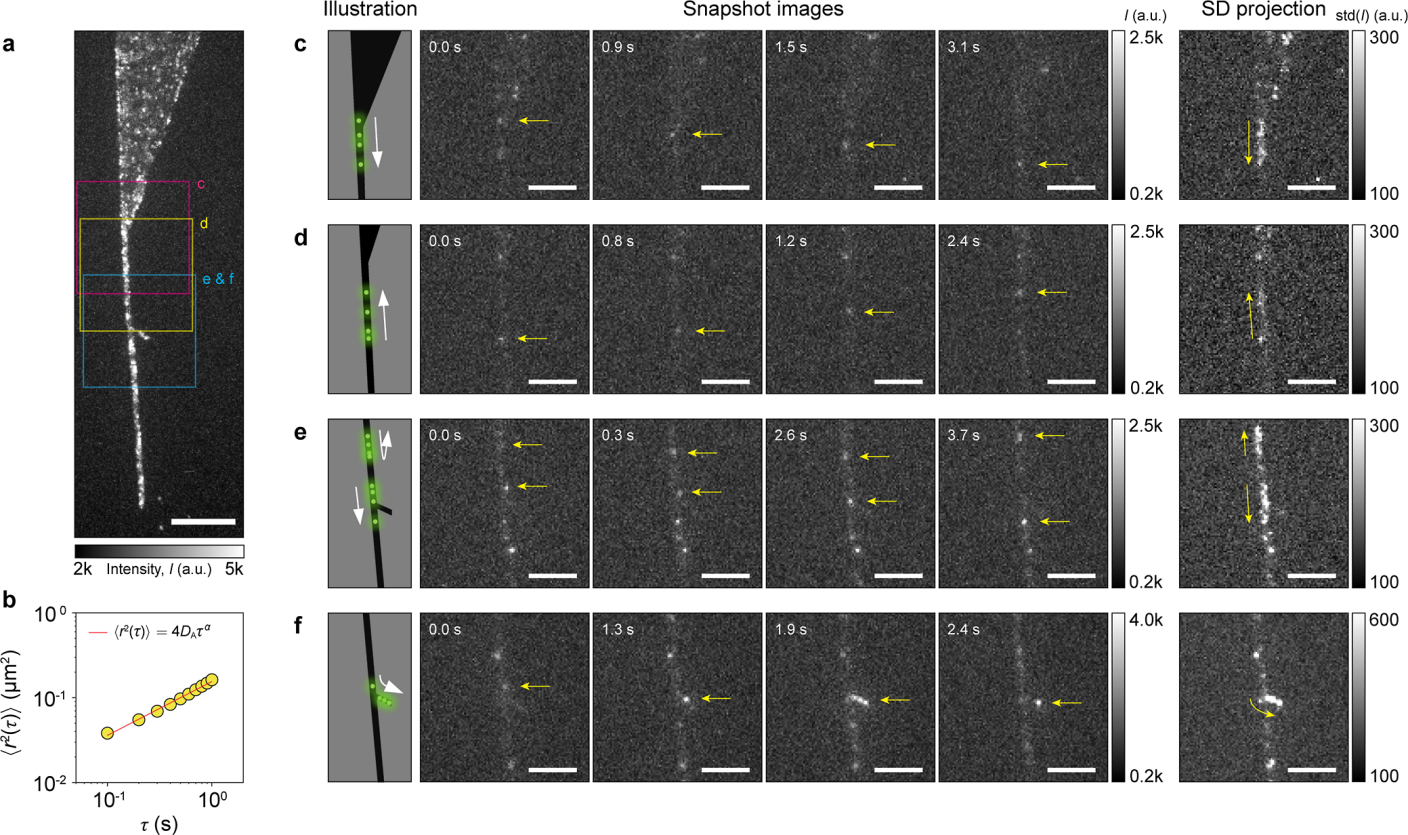
Surface diffusion of ssDNA molecules on a hBN ribbon. (a) A time-based maximum projection of 3500 epifluorescence microscopy images, showing 16 nt ssDNA molecules on a hBN surface. The hBN flake consists of three parts, a large reservoir (top), a narrow channel (bottom), and a *y*-junction branch (middle). The scale bar is 10 μm. (b) The mean square displacement (MSD) as a function of the lag time, averaged over 292 trajectories. (c–f) Snapshots of ssDNA injection from the reservoir to the channel (c), linear motion along the channel (d), straight (e) and bending (f) movements at the *y*-junction. In the last column, the images show the standard deviation projection of the image stack over time, where the pixels with large intensity changes across the image stack are brighter. The presence of bright patches indicates the presence of mobile DNA molecules throughout the respective time periods (3.1, 2.4, 3.7, and 2.4 s for (c–f), respectively). The scale bars in (c–f) are 5 μm.

Our study shares a conceptual foundation with recent theoretical publications on biomolecule transport in nanometer-scale channels on 2D materials.^19,23,24,27,66^ However, while these studies specifically examined narrow channels of a few nanometers, where external electric fields and strong steric confinement can induce significant conformational changes in the biomolecules, our hBN ribbon structures ranging from hundreds of nanometers to micrometers in width create a pseudo-1D environment that permits ssDNA to retain its conformational flexibility. Despite these differences, the overarching conclusion that 2D surfaces can serve as platforms for directed biomolecular transport is consistent among these studies, underscoring the potential of hBN surfaces for controlled molecular transport applications.

## 3 Conclusions

We investigated the dynamics of DNA molecules on hBN surfaces using a multifaceted approach, yielding quantitative and mechanistic insights at single-molecule level from experiments and numerical simulations. We showed that ssDNA molecules can diffuse over untreated hBN surfaces for substantial periods without binding to contaminants. Comparing our single-molecule tracking results to complementary computational studies provided insights into the mechanisms of surface diffusion and confinement of ssDNA molecules as well as ssDNA–hBN interactions. Strikingly, our study revealed reduced mobility at step edges on hBN surfaces, resulting in the confinement of ssDNA molecules within the boundaries of the domains. These findings open avenues for constructing 2D nanofluidic devices featuring tightly constrained channels, which will enable directional, one-dimensional motion of biomolecules, whether through surface diffusion or external stimuli.

Atomically flat 2D surfaces have the potential to address fundamental biophysical questions and offer high-resolution sensing in confined environments. They neither require biochemical tethering strategies to bind molecules to a surface, nor do they rely on complex nanofabrication protocols to achieve confinement. Our systematic investigation of DNA transport on hBN surfaces provides insights into the fundamental mechanisms governing biomolecular behavior on 2D materials, particularly the roles of surface defects and geometrical constraints in molecular diffusion. This understanding of basic molecular transport mechanisms on 2D surfaces could contribute to the development of future molecular manipulation strategies.

## 4 Methods

### 4.1 Preparation of the hBN Surface

Pristine hBN crystals provided by T. Taniguchi and K. Watanabe at the National Institute for Materials Science (Tsukuba, Japan) were exfoliated onto borosilicate glass coverslips (No. 1.5, VWR, 170 μm thick) using the mechanical exfoliation method with 3 M Scotch tape.^67^ The coverslips were cleaned through initial sonication in Milli-Q (MQ) water for 5 min, followed by sequential sonication in organic solvents (acetone, methanol, and IPA, 15 min each) with intermediate MQ water sonication for 5 min between each solvent step. The cleaning was completed with KOH treatment (1 M, 30 min) and final MQ water sonication. After drying under nitrogen, high-quality hBN flakes were exfoliated onto the freshly cleaned coverslips within 6 h (typically 1–2 h) prior to measurement.

### 4.2 DNA and Buffers

Fluorescent dye (Cy3)-labeled single-stranded DNA (ssDNA) samples were purchased from Ella Biotech and used without further purification. We used Cy3 because it is well established for single-molecule imaging and thoroughly characterized in our laboratory, ensuring a reliable baseline for data interpretation. Five ssDNA samples with different lengths were used in this study (7 nt, Cy3-CCTCCTC; 15 nt, AGATTTTTTTTTTTT-Cy3; 16 nt, Cy3-TTTTTTTTTTTTTTTT; 35 nt, AGATTTTTTTTTTTTTTTTTTTTTTTTTTTTTTTT-Cy3; 100 nt, AATGATACGGCGACCACCGAGATCTACACTCTTTCCCTACACGACGCTCTTCCGATCTACGTATCACGAAAAAAAAATCXCGTATGCCGTCTTCTGCTTG), where X denotes amine-modified thymine base. The ssDNA samples were stored at 10 nM concentration in a buffer of 10 mM TrisHCl pH 8.0 and 50 mM NaCl. For each measurement, the ssDNA was diluted to a desired concentration immediately before applying it to the hBN surface. We intentionally excluded oxygen scavenging and photostabilizing reagents from our buffer, as their components, consisting of proteins and chemicals at micromolar to millimolar concentrations, could interact with the hBN and potentially crowd the surface. Such interactions could complicate our interpretation of diffusion behavior and surface interactions. To avoid evaporation of the droplet on the coverslip, another coverslip was placed above the bottom coverslip with a ring or hollow square-shaped PDMS film (1 mm thickness) as a spacer (see Figure 1a).

### 4.3 Epifluorescence Microscopy Setup

To measure single-molecule fluorescence signals, a home-built epifluorescence microscope setup built around a commercial inverted microscopy body (Eclipse Ti2, Nikon) equipped with a high numerical aperture objective lens (60×, NA1.2, water immersion, Nikon) was used (Figure 1a). The beam size of a 532 nm laser CW diode-pumped solid-state laser (06-DPL 532 nm, Cobolt) was enlarged by using a telescope and focused on the back focal plane of the objective lens for widefield excitation in epi-fluorescence mode. To optimize conditions for single-molecule imaging and minimize photobleaching, we used a relatively low laser intensity (∼100 W/cm^2^ at the hBN surface), typically achieving photon counting rates of 400–450 Hz from individual fluorescent dyes. The Cy3 fluorescence signal was filtered by using a laser-blocking filter (ZET532/640m, Chroma Technology) and single-molecule fluorescence images were recorded by using an EMCCD (Ixon Ultra, Andor).

### 4.4 Single-Molecule Fluorescence Measurement

For the single-molecule fluorescence measurement on hBN surfaces, we manually scanned for an hBN flake using a white light source. Once a flake was located within the field of view, we applied a 5 μL droplet of blank buffer (10 mM TrisHCl pH 8.0 and 50 mM NaCl) to the hBN flake and incubated it for 2 min. Following the incubation, we evaluated the level of background noise and contamination under 532 nm laser excitation. Afterward, we added another 5 μL droplet of buffer solution (10 mM TrisHCl pH 8.0 and 50 mM NaCl) containing 1 pM ssDNA molecules to the existing droplet. After 2 min incubation in a dark room to allow the contents of the two droplets to mix, the fluorescence signals were recorded with a frame time of 100 ms.

The concentration of ssDNA selected for the adsorption test was determined based on the reliability of the data and molecular crowding. Higher concentrations, such as 100 pM, complicated the identification and quantification of individual spots due to excessive overlap, rendering accurate counting unfeasible. Conversely, at lower concentrations (e.g., femtomolar levels), the time required to observe a measurable increase in spot numbers was impractically long. Additionally, at these lower concentrations, differentiating between ssDNA molecules and contaminants within the buffer became challenging, as the adsorption rates of both were similarly low.

For the observations presented in Figure 1, the laser shutter was opened only for brief intervals, specifically 2–3 s every 30 s, to reduce fluorophore photobleaching. All the measurements were carried out at room temperature.

### 4.5 Single-Molecule Tracking

Single-molecule images were analyzed with the TrackMate plugin in the Fiji software.^68,69^ Positions of individual fluorescent spots were determined by using differences of Gaussian algorithm combined with a quadratic fitting scheme with subpixel resolution.^70^ To eliminate the interference of highly luminous spots near the flake boundary, the exterior of the boundary, beginning from its inner edge, was cropped out. Furthermore, a threshold for the sum intensity was applied to eliminate potential false-positive spots along the border, which might arise due to the stark contrast between the cleared and uncleared regions. Once the positions of individual molecules were ascertained, their trajectories across consecutive frames were determined using the linear assignment problem mathematical framework.^71^ The trajectory of a particle was determined by linking the particle’s position within consecutive frames if it is found in the next frame within 5 pixels (0.88 μm), as the probability of having a 2D displacement exceeding this value is less than 1% according to the analysis shown in Figure 2e.

Typical photon counts measured in our system ranged from 40 to 45 photons per 100 ms, with background counts of ∼20 photons. Both pixel intensity values, *I*_pixel_, and background counts were determined by fitting the measured fluorescence spots with a 2D Gaussian function. To convert pixel intensity values into photon counts, we used the following equation that accounts for the electron-multiplying (EM) gain and CCD sensitivity of the EMCCD camera

$$N_{photon}=I_{pixel}\times CCD\;sensitivity÷EM\;gain$$

where the CCD sensitivity and the EM gain correspond to 9.41 (electrons per A/D counts) and 200 (per photon), respectively. According to the approach described by Thompson et al.,^60^ the precision of particle position determination was estimated as 0.096 μm, based on the Cramér–Rao lower bound.

It should be noted that Cy3 fluorescence molecule itself also binds to hBN surfaces and exhibits significantly faster movement on the hBN surface compared to ssDNA molecules. Due to this rapid movement, the Cy3 molecules appear blurred rather than as separate spots under our experiment conditions of 100 ms frame time (Figure S7 and Movies S10, S11, S12 and S13). As a result, Cy3 molecules are naturally excluded during the spot identification process in single-molecule tracking analysis, thus avoiding any influence on the analysis of ssDNA diffusion. A similar motion blur effect also influences the apparent brightness of dye-labeled DNA molecules depending on their speed. Nevertheless, we obtained sufficient photons for reliable single-molecule tracking even from the shortest, and therefore fastest, DNA strands.

### 4.6 Diffusion Analysis

The mean squared displacement (MSD) for a given lag time τ = *n*Δ*t* was calculated for each individual trajectory using $\left\langle r^{2}\left(\tau \right)\right\rangle =\frac{1}{l-n}\overset{l-n}{\underset{i=1}{\sum }}||\mathrm{r⃗}(i+n)-\mathrm{r⃗}(i)|{|}^{2},$ where *r⃗*(*k*) = [*x*(*k*),*y*(*k*)], with *x*(*k*) and *y*(*k*) denoting the coordinates of the molecule at the frame index *k*.^61,72^ Here, *l*, Δ*t*, and *n* denote the trajectory length, the frame time, and a positive integer, respectively. To improve the reliability of our diffusion measurements, we calculated the MSD only from trajectories longer than 20 frames (2 s), following a commonly used approach in MSD analysis, where short tracks are excluded to reduce noise and ensure accurate estimation.^72^ The track length distributions show no significant dependence on DNA length (Figure S8), indicating consistent desorption behavior across all lengths. Furthermore, the total spot number exhibits a smooth and monotonic decay trend over time for all DNA lengths (Figure S9), indicating a uniform photobleaching probability across frames. These results support that excluding short tracks does not introduce systematic bias related to DNA length.

The apparent diffusion coefficient *D*_A_ and the diffusion exponent α of the ensemble average of the MSD were determined by a linear fitting of the MSD plot using a 2D diffusion equation of ⟨*r* ^2^(τ)⟩ = 4*D*_A_τ^α^. At a specific frame index *k* with a time window *T*, the temporal MSD for the lag time *n*Δ*t* was calculated as^73^ $\left\langle r_{temp}^{2}\left(n\Delta t\right)\right\rangle =\frac{1}{T-n}\overset{T-n}{\underset{i=1}{\sum }}||\mathrm{r⃗}(k-1+i+n)-\mathrm{r⃗}(k-1+i)|{|}^{2}.$ From the temporal MSD, the temporal diffusion coefficient *D*_A,temp_ were derived: $D_{A,temp}=\sum_{j=1}^{T-1}w\left(j\right)\frac{r_{temp}^{2}\left(j\Delta t\right)}{4\times j\Delta t}$ , where the weighting factor ${w\left(j\right)}^{-1}=\frac{j\left(2j^{2}+1\right)}{T-j+1}$ corresponds to the relative variance of ⟨*r*_temp_^2^(*j*Δ*t*)⟩.^61^

### 4.7 MD Simulation

#### 4.7.1 MD Simulation Methods

All MD simulations were performed using NAMD2.14,^74^ the CHARMM36 parameter set^75^ for DNA, TIP3P water model^76^ and the CUFIX corrections^77^ to ion-nucleic acid interactions. The nonbonded interactions for B and N atoms in the hBN surface were set up according to previous work.^78^ The hydrogen repartitioning scheme was used to achieve a 4 fs time-step^79^ with local and long-range interactions computed every 4 fs. All short-range nonbonded interactions were cut off starting at 1 nm and completely cut off by 1.2 nm. Long-range electrostatic interactions were evaluated using the particle-mesh Ewald method^80^ computed over a 0.1 nm spaced grid. SETTLE^81^ and RATTLE82 ^82^ algorithms were applied to constrain covalent bonds to hydrogen in water and in nonwater molecules, respectively. The temperature was maintained at 295 K using a Lowe–Andersen thermostat^83^ with a cutoff radius of 2.7 Å for collisions and the rate of collisions at 50 ps^–1^. Energy minimization was carried out using the conjugate gradients method.^84^ Initial equilibrations, ∼20 ns, were performed in the constant pressure ensemble, using a Nose–Hoover Langevin piston with a period and decay of 2000 and 1000 fs, respectively.^85^ Subsequently, all production simulations were performed in the constant volume ensemble. Throughout all simulations, the hBN surface atoms were fixed using harmonic position restraints with a scaling factor of 1 kcal mol^–1^ Å^–2^. Atomic coordinates were recorded every 9.6 ps. Visualization and analysis were performed using VMD and MDAnalysis.^86,87^

#### 4.7.2 Preparation of the MD Simulation System

An hBN sheet consisting of 3 layers, was generated using VMD’s^86^ nanotube plugin. The 35 and 60 nt ssDNA were built using Avogadro.^88^ Shorter sequences were generated by morphing residues from the 35 or 60 nt structures to match the desired ssDNA sequence using VMD’s psfgen plugin. The DNA molecule was then placed close to the hBN surface and submerged in a 50 mM NaCl solvent, using the solvate and autoionize plugins in VMD.^86^ Required number of ions were determined by considering molality (mol kg^–1^). All systems were simulated with hexagonal periodic boundary conditions. Atoms on the boundary were covalently bonded across the periodic lattice, to create an effectively infinite hBN surface. For the hBN surfaces, three distinct types were simulated: (i) a perfectly flat surface, (ii) a surface with a single step edge, and (iii) a surface with atomic defects. The hBN surface with a single step edge was constructed by removing one-half of the atoms on the topmost hBN layer. The partial charge on atoms present on the defect boundary was set zero, to avoid unphysical pinning of nucleotides at the boundary. The hBN surface with atomic defects were created by random removal of B and N atoms on the topmost hBN surface. Here, the stoichiometry between B and N atoms, and hence the charge, was preserved during the removal of atoms.

#### 4.7.3 Summary of MD Simulation Systems

In total, we simulated ten independent systems, each differing by the ssDNA sequence, the nucleotide count, and the presence of defects on the hBN surface. Four systems contained a poly­(dAdT) DNA homopolymer c made of either 7, 15, 35, or 64 nucleotides were simulated on a perfectly flat hBN surface, i.e. devoid of any defects for ∼300 ns each. These systems contained approximately 250k, 250k, 390k and 700k atoms respectively, and each of these were simulated for ∼300 ns. Another 250k-atom system contained a poly­(dAdT)_12_ strand and an hBN surface having a single step edge and was simulated for ∼500 ns. Another three systems contained a poly­(dAdT)_35_ strand and an hBN surface with atomic defects produced by randomly removing 1, 0.2 or 0.1% of both B and N atoms. Finally, two separate ∼250k-atom systems contained either a poly­(dA)_25_ or poly­(dT)_25_ strand and were each simulated in the presence of a smooth hBN surface for ∼300 ns.

### 4.8 MC Simulation

As illustrated in Figure 4a, the 2D diffusion was modeled with the following conditions: the DNA molecules diffuse on a finite square surface with area *L*_d_^2^, with a free-diffusion coefficient (*D*_0_) obtained from MD simulations of a perfectly flat hBN surface. To ensure sampling efficiency and accuracy, we used a 1 ns time step—based on the observed largest diffusion coefficient of ∼749 μm^2^/s for 7 nt ssDNA, which translates to the root-mean-square displacement of less than 1 nm in 1 ns—thereby preventing molecules from skipping over defects within a single step.

To model the observed temporary trapping of molecules at defects, two more parameters other than *D*_0_ and *L*_d_ were introduced: (i) a trapping probability *P*_tr_ per simulation step of 1 ns and (ii) the trap time τ_t_. The simulation allowed molecules to be in either moving or trap state at each time step, with all molecules initially in the moving state. The state transition was governed by a random number generated between 0 and 1 each time step; if this number was lower than *P*_tr_, the molecule transitioned to the trap state. During the trap state, molecules remained stationary for a trap time τ_t_. Once the trap duration ended, molecules resumed moving.

Each simulation spanned up to 2 s of simulated time and tracked 100 molecules, although in certain parameter regimes (e.g., trapping probability *P*_tr_ = 0% and 0.01%), we reduced the number of molecules for computational feasibility. The trap times τ_t_ were randomly generated from a log–normal probability density function distribution $\Psi \left(\tau_{t}|\mu ,\sigma \right)=\frac{\tau_{0}}{\tau_{t}\sigma \sqrt{2\pi }}e^{\left\{-{\left(\ln\left[\tau_{t}/\tau_{0}\right]-\mu \right)}^{2}/2\sigma^{2}\right\}}$ , with mean μ, variance σ^2^, and τ_0_ = 1 s. To avoid an unmanageable number of 1 ns steps, we advanced the simulation by sampling this log-normal trap time during trapping events.

From the analysis of the trap time distribution we observed experimentally (Figure S6a–c), we determined μ = −8.0 and σ = 2.25 for the 7 nt DNA, as shown in Figure S6d. In the case of 15 nt DNA, the values were found to be μ = −8.0 and σ = 2.55, as presented in Figure S6e. Once a comprehensive trajectory recorded with 1 ns time intervals is generated, trajectories were then extracted from the original trajectory and reconstructed for various observation frame time Δ*t* ranging from 1 ns to 100 ms. Using the trajectories reconstructed according to the Δ*t*, the mean squared displacement (MSD) as a function of the lag time τ for each molecule was calculated and the average MSD, ⟨*r* ^2^(τ)⟩, was then derived by averaging out the MSDs across all molecules, which allowed us to compute the apparent diffusion coefficients *D*_A_ and diffusion exponents α for each observation frame time Δ*t* using the equation ⟨*r* ^2^(τ)⟩ = 4*D*_*A*_τ^α^. As shown in Figure S10, the violin plots of *D*_A_ and α at the frame time of 100 ms (our measurement condition) show narrow distributions around the mean values, indicating that our MC approach converges reliably under various trapping probabilities *P*_tr_ and domain sizes *L*_d_.

## Supplementary Material





























## Notes

AFM images of hBN surfaces with step edges; MD simulation of ssDNA diffusion on a perfectly flat hBN surface; MD simulation of multiple sequences of 25 nt ssDNA on a perfectly flat hBN surface; MD simulation of ssDNA diffusion on a hBN surface containing a single step defect; MD simulation of ssDNA diffusion on hBN surfaces containing different density of atomic defects; Analysis and modeling of trap times; Single-molecule observation of Cy3 and ssDNA on the hBN surface; Track length distribution for ssDNA of varying lengths; Temporal evolution of visible ssDNA spot counts; Analysis of diffusion parameters simulated from 100 molecules (PDF)
Time-lapse fluorescence microscopy of 7 nt ssDNA diffusion on a hBN surface (MP4)
Time-lapse fluorescence microscopy of 15 nt ssDNA diffusion on a hBN surface (MP4)
Time-lapse fluorescence microscopy of 35 nt ssDNA diffusion on a hBN surface (MP4)
Time-lapse fluorescence microscopy of 100 nt ssDNA diffusion on a hBN surface (MP4)
MD simulation of ssDNA diffusion on a perfectly flat hBN surface (MP4)
MD simulation of ssDNA diffusion on a hBN surface containing a single step defect (MP4)
MD simulation of ssDNA diffusion on a hBN surface containing atomic defects (MP4)
MD simulation of ssDNA diffusion on a hBN surface containing atomic defects (a zoom-in version) (MP4)
Time-lapse fluorescence microscopy of 16 nt ssDNA diffusion on a hBN ribbon (MP4)
Initial observation of a hBN surface with blank buffer (MP4)
Following up with Cy3 at 10 pM on the hBN surface (MP4)
Increasing concentration to Cy3 at 100 pM on the hBN surface (MP4)
Introducing 7nt ssDNA at 100 pM on the hBN surface (MP4)
