# Supplementary material for: Diffusion of DNA on Atomically Flat 2D Material Surfaces
Source: ACS Nano. 2025 Jun 5;19(23):21307–18. doi: 10.1021/acsnano.4c16277 (PMC12177950; doi:10.1021/acsnano.4c16277)
Supplement: Supplementary file 1 [file nn4c16277_si_001.pdf]

# Supplementary Information

# Diffusion of DNA on Atomically Flat 2D Material Surfaces

Dong Hoon Shin,[1,2,3,§] Sung Hyun Kim,[1,4,5,§] Kush Coshic,[6] Kenji Watanabe,[7] Takashi Taniguchi,[7] Gerard J. Verbiest,[2] Sabina Caneva,[2] Aleksei Aksimentiev,[6,8] Peter G. Steeneken,[1,2,*] and Chirlmin Joo[1,4,9,*]

[1]Kavli Institute of Nanoscience Delft, Lorentzweg 1, 2628 CJ Delft, The Netherlands

[2]Department of Precision and Microsystems Engineering, Delft University of Technology, Mekelweg 2, 2628 CD Delft, The Netherlands

[3]Department of Electronics and Information Engineering, Korea University, Sejong 30019, Republic of Korea

[4]Department of Bionanoscience, Delft University of Technology, 2629 HZ Delft, The Netherlands

[5]New and Renewable Energy Research Center, Ewha Womans University, Seoul 03760, Republic of Korea

[6]Center for Biophysics and Quantitative Biology, University of Illinois at Urbana-Champaign, Urbana, Illinois 61801, United States

[7]Advanced Materials Laboratory, National Institute for Materials Science, Tsukuba 305-0044, Japan

[8]Department of Physics and Beckman Institute for Advanced Science and Technology, University of Illinois at Urbana-Champaign, Urbana, Illinois 61801, United States

[9]Department of Physics, Ewha Womans University, Seoul 03760, Republic of Korea

[§]These authors contributed equally to this work.

**This PDF file includes:**

Supplementary Figures. S1 to S10

Figure S1. AFM images of hBN surfaces with step edges.
Figure S2. MD simulation of ssDNA diffusion on a perfectly flat hBN surface.
Figure S3. MD simulation of multiple sequences of 25 nt ssDNA on a perfectly flat hBN surface.
Figure S4. MD simulation of ssDNA diffusion on a hBN surface containing a single step defect.
Figure S5. MD simulation of ssDNA diffusion on hBN surfaces containing different density of atomic defects.
Figure S6. Analysis and modelling of trap times.
Figure S7. Single-molecule observation of Cy3 and ssDNA on the hBN surface.
Figure S8. Track length distribution for ssDNA of varying lengths (7, 15, 35, and 100 nt) under identical measurement conditions, including laser power and temperature.
Figure S9. Temporal evolution of visible ssDNA spot counts for varying lengths (7, 15, 35, and 100 nt) under identical measurement conditions, including laser power and temperature.
Figure S10. Analysis of diffusion parameters simulated from 100 molecules.

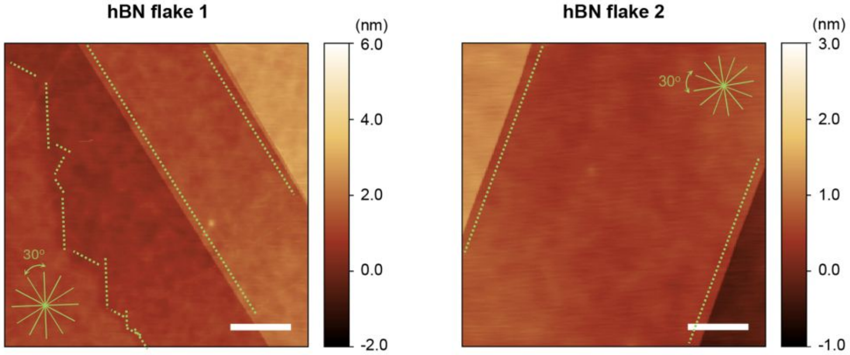


**Figure S1. Atomic force microscopy (AFM) images of hBN surfaces with step edges.** The AFM images clearly show terraces and atomic steps, which potentially form domain boundaries for DNA diffusion on the hBN surfaces. The edges display 30-degree angles between them (indicated by green dotted lines), demonstrating that they align with the armchair and zig-zag directions of the hexagonal crystal structure. The scale bars are 2 μm.

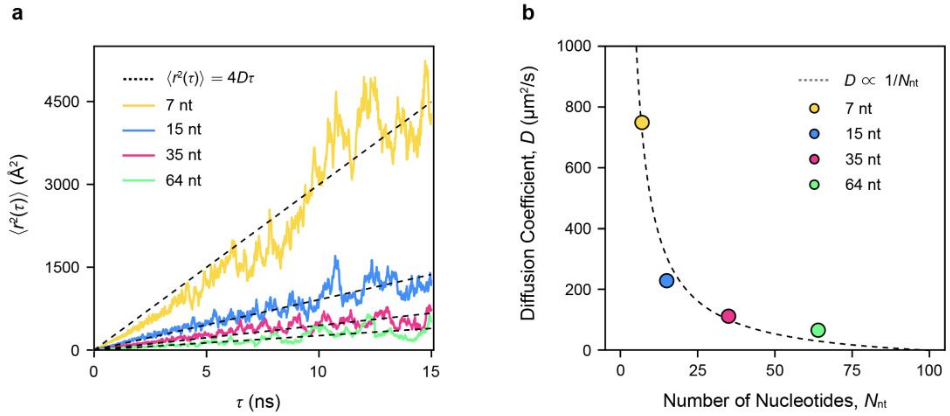


**Figure S2. MD simulation of ssDNA diffusion on a perfectly flat hBN surface.** (a) Mean squared displacements (MSD) of ssDNA of four different lengths. The ssDNA sequences used in the simulation were identical to those in the experiments. Diffusion coefficients $D$ of the ssDNA molecules with lengths of 7, 15, 35, and 64 nt were found to be 749, 228, 111, and 65 μm$^2$/s, respectively. These values were determined from the slope of MSD plots using the equation $\langle r^2(\tau)\rangle = 4D\tau^{\alpha}$, where $\langle r^2(\tau)\rangle$ and $\tau$ represent the MSD and the lag time, respectively. The diffusion exponent α is set to 1, indicating normal diffusion. (b) The diffusion coefficients are plotted as a function of the number of nucleotides $N_{nt}$. The dashed line is a fit to the data with $D = C \times 1/N_{nt}$.

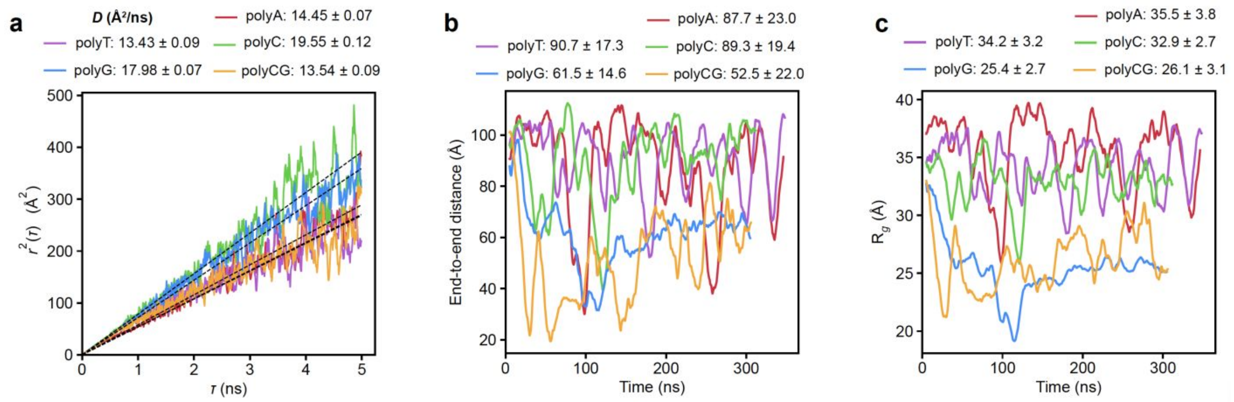


**Figure S3: MD simulation of multiple sequences of 25 nt ssDNA on a perfectly flat hBN surface.** (a) The MSD plots of five types of DNA simulated. The diffusion coefficients were determined from the slopes. (b) Representative time courses of the end-to-end distance, defined as the instantaneous distance between O5' and O3' atoms of the respective 5' and 3' ends of the ssDNA. (c) Evolution of the radius of gyration during the simulation. For visual clarity a running average over a window of 0.02 ns is plotted in b and c.

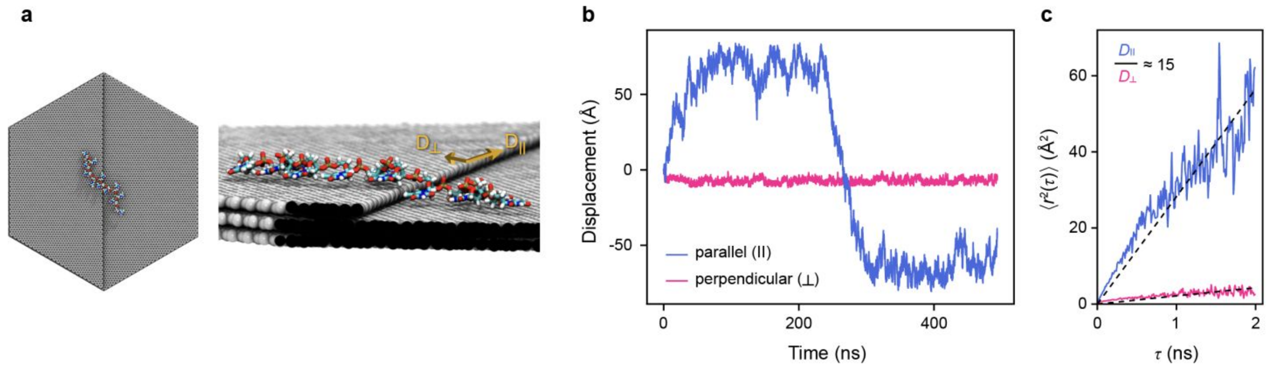


**Figure S4. MD simulation of ssDNA diffusion on a hBN surface containing a single step defect.** (a) Side (left) and top (right) view of a 12 nt polyT ssDNA molecule positioned on a step defect. (b) Displacement of the centre-of-mass (CoM) of the molecule projected parallel (blue) and perpendicular (pink) to the step edge. (c) Projected MSDs calculated for the directions parallel (blue) or perpendicular (pink) to the step edge. The diffusion coefficient of the parallel direction to the step edge was approximately 15 times larger than that of the perpendicular direction.

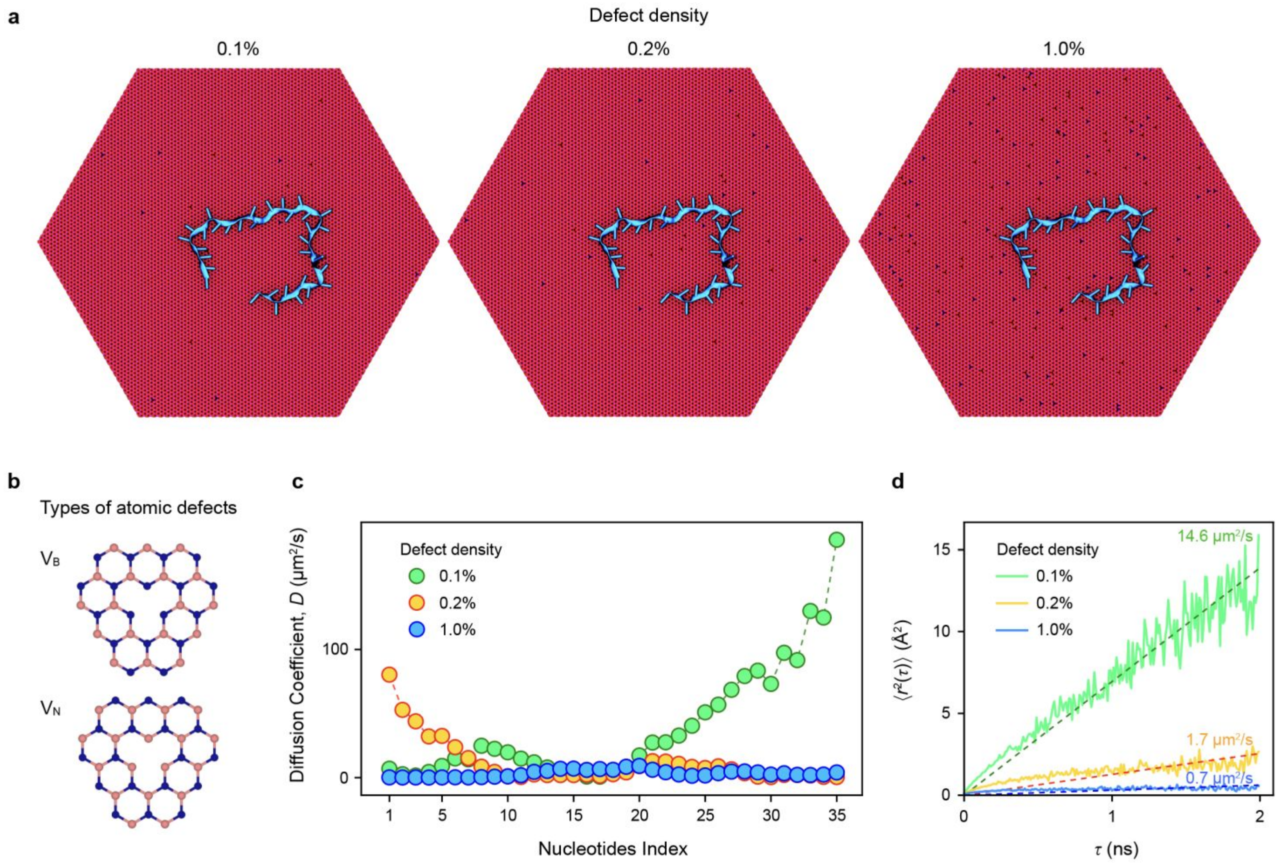


**Figure S5. MD simulation of ssDNA diffusion on hBN surfaces containing different density of atomic defects.** (a) Initial setup of three simulation systems, each characterized by different defect densities (0.1, 0.2, and 1.0%). In these simulations, a 35 nt ssDNA molecule (blue) was positioned on the hBN surfaces (red) with randomly placed defects (dark spots). Water and ions in the system are not depicted for clarity. (b) Types of atomic defects employed in the simulation systems: boron vacancy ($V_B$) and nitrogen vacancy ($V_N$). (c) Diffusion coefficients of individual nucleotides in the 35 nt ssDNA molecule for the three systems. The dynamics of the ssDNA molecule on the hBN surface with 0.1% defect density can be observed in Movie S7 and S8. (d) The MSD of the CoM motion of the entire DNA molecule for each system, demonstrating a clear relationship between defect densities and diffusion coefficients.

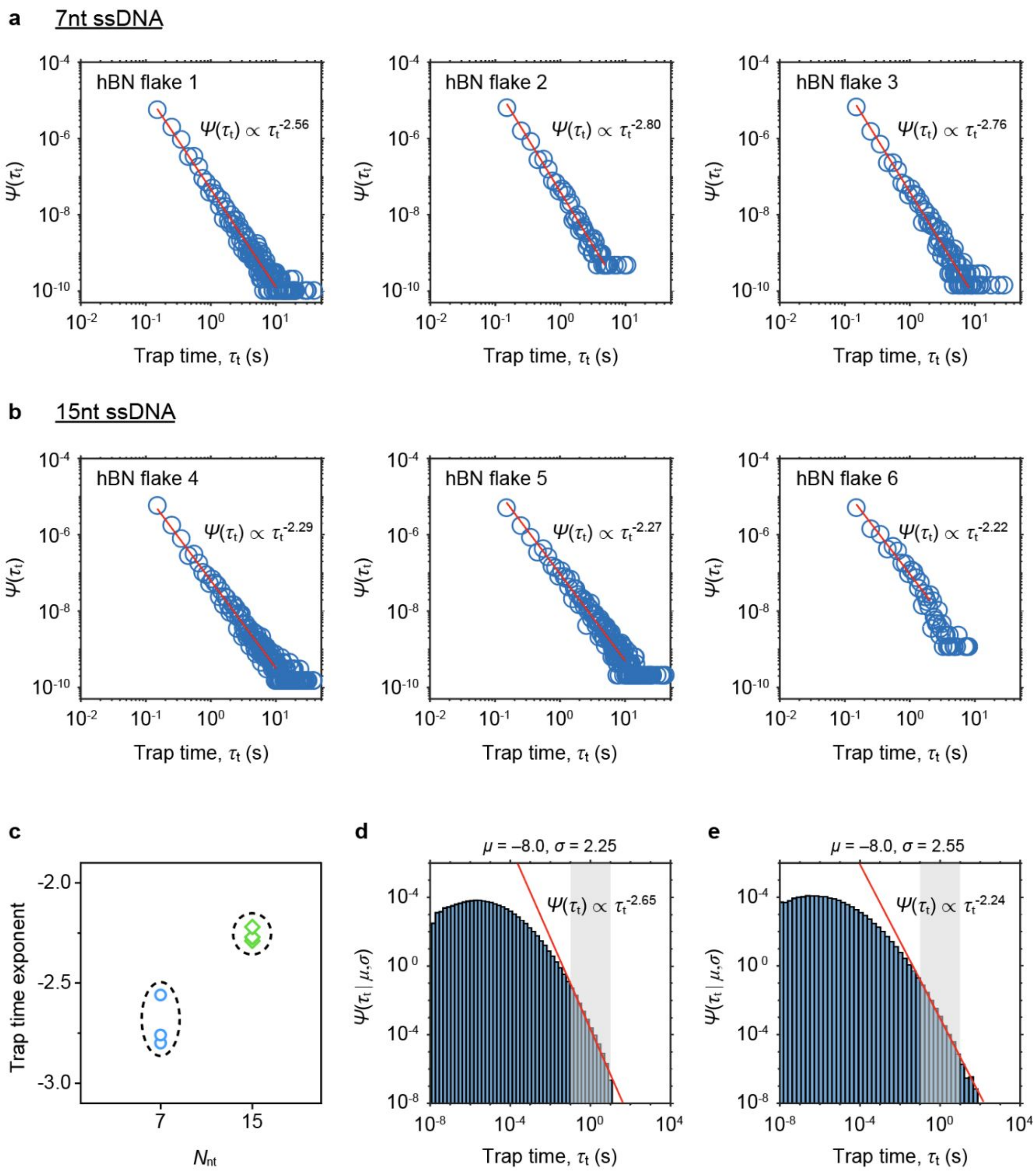


**Figure S6. Analysis and modelling of trap times.** (a) and (b) display the distributions of trap times for 7 nt and 15 nt ssDNAs, respectively, alongside power-law fits. These were determined from single molecule fluorescence microscopy measurements on various hBN surfaces. Trap times were calculated as intervals between significant movements, identified when displacements between successive positions exceeded a threshold of 0.176 μm² (one EMCCD pixel size in our measurement setup). The

trap time distributions were normalized to form probability distributions, with an offset for clarity, and a linear fit was applied to log-transformed data to establish a power-law relationship. (c) The trap time exponents of both 7 nt and 15 nt ssDNAs. The 15 nt ssDNA exhibits longer tails compared to the 7 nt ssDNA, indicating a broader range of trap times. (d) One million random numbers generated from a log-normal distribution with varying mean ($\mu$) and standard deviation ($\sigma$) of logarithmic values to find the mean ($\mu$) and standard deviation ($\sigma$) of logarithmic distribution that matches the experimentally obtained trap time distribution. Histograms were created and normalized as probability density functions using logarithmically spaced bins. These were fitted within the 0.1-10 s range (grey shaded area), corresponding to the experimentally observable range, to derive power-law parameters. The distribution parameters for 7 nt and 15 nt ssDNAs were $\mu$ = -8.0 and $\sigma$ = 2.25, and $\mu$ = -8.0 and $\sigma$ = 2.55, respectively.

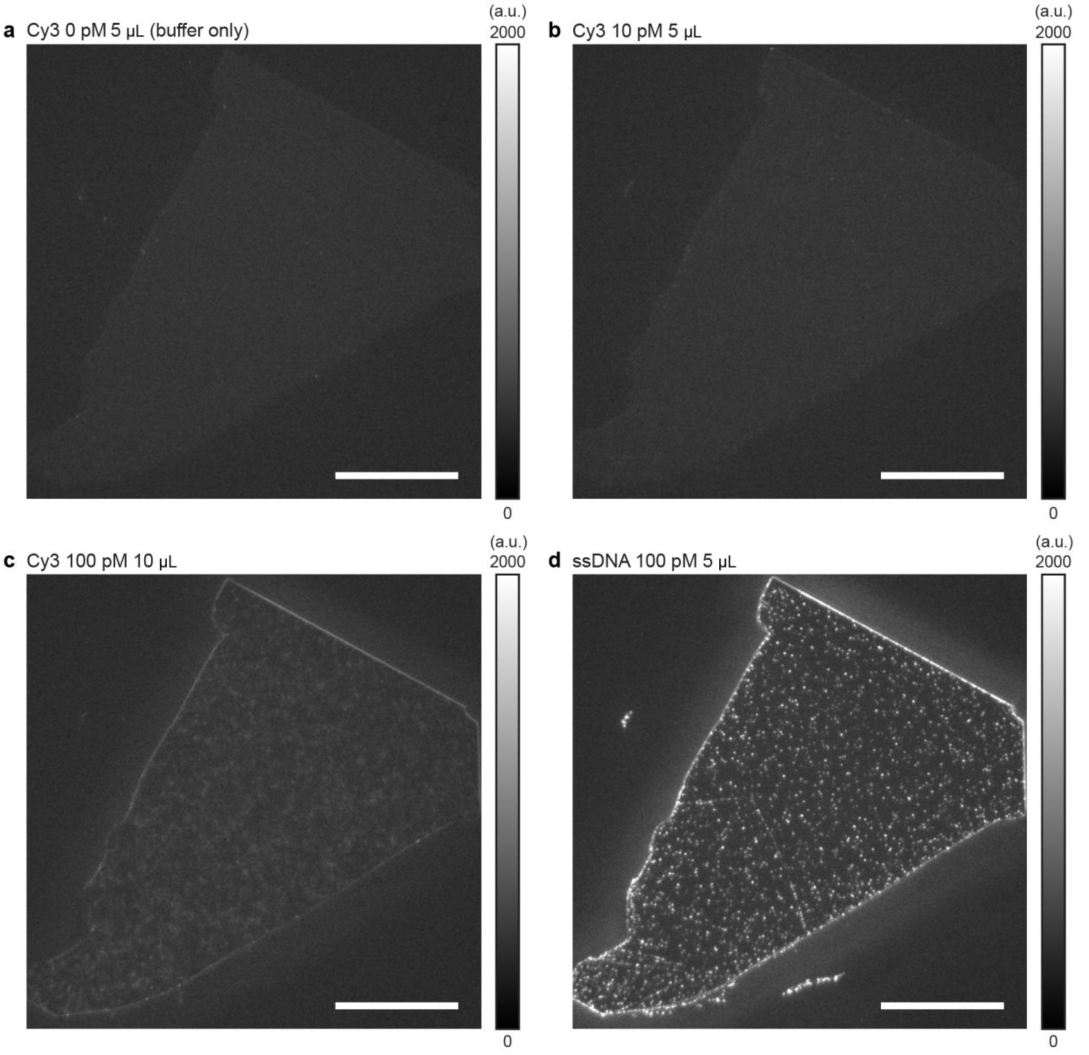


**Figure S7. Single-molecule observation of Cy3 and ssDNA on the hBN surface.** The series of fluorescence microscopy images shows Cy3 fluorescence molecules and Cy3-labelled ssDNA molecules adsorbed on the hBN surface after the addition of a 5 μL droplet containing neither ssDNA nor Cy3 (a), 5 μL of 10 pM Cy3 (b), 10 μL of 100 pM Cy3 (c), and 5 μL of 100 pM 7nt ssDNA (d), respectively (Movie S10-S13). The images were captured using a frame time of 100 ms. The scale bars represent 20 μm.

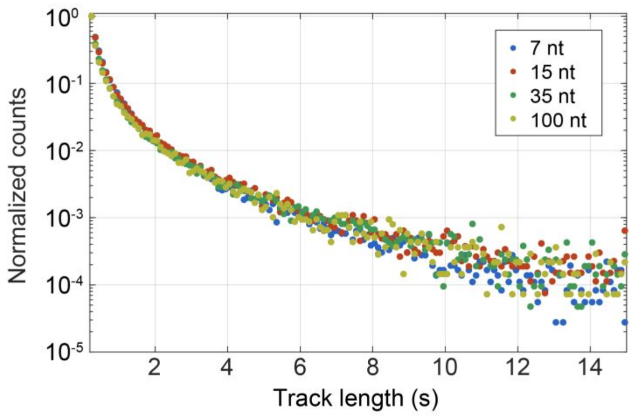


**Figure S8. Track length distribution for ssDNA of varying lengths (7, 15, 35, and 100 nt) under identical measurement conditions, including laser power and temperature.** The track length distributions (*i.e.*, track lifetimes) exhibit no significant dependence on DNA length, suggesting that both desorption and photobleaching processes are homogeneous in time across all DNA lengths.

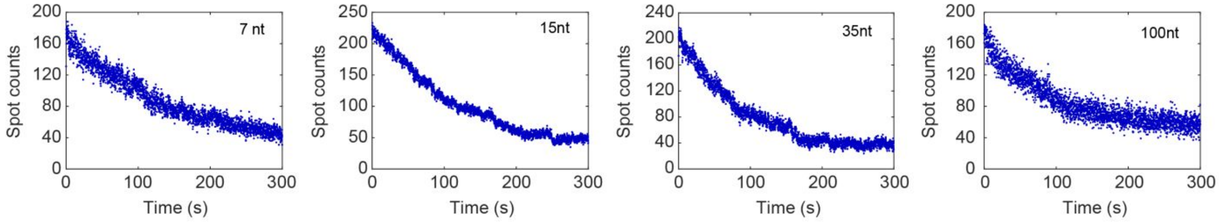


**Figure S9. Temporal evolution of visible ssDNA spot counts for varying lengths (7, 15, 35, and 100 nt) under identical measurement conditions, including laser power and temperature.** The total spot number exhibits a smooth and monotonic decay trend over time for all DNA lengths.

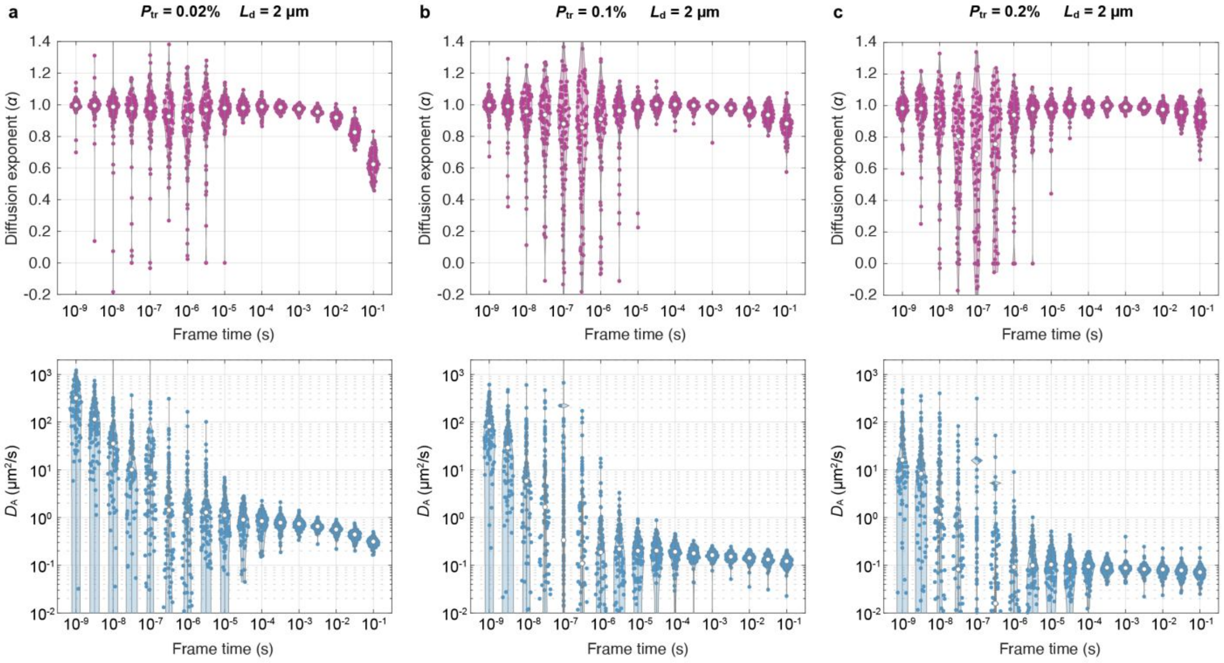


**Figure S10. Analysis of diffusion parameters simulated from 100 molecules.** Violin plots of the apparent diffusion coefficient ($D_A$, bottom panel) and the diffusion exponent ($\alpha$, top panel)) from 100 different molecules under different simulation conditions. (a) $P_{tr}$ = 0.02%, $L_d$ = 2 μm, (b) $P_{tr}$ = 0.1%, $L_d$ = 2 μm, and (c) $P_{tr}$ = 0.2%, $L_d$ = 2 μm.
